# Extract of *Marsdenia tenacissima* (Roxb.) Moon [Apocynaceae] Suppresses Hepatocellular Carcinoma by Inhibiting Angiogenesis

**DOI:** 10.3389/fphar.2022.900128

**Published:** 2022-06-30

**Authors:** Yating Pan, Xinyi Liao, Lili Yang, Chunlei Zhang, Jue Wang, Peiyong Zheng, Guanzhen Yu, Haiyan Song

**Affiliations:** ^1^ Institute of Digestive Diseases, Longhua Hospital, Shanghai University of Traditional Chinese Medicine, Shanghai, China; ^2^ Department of Oncology, Longhua Hospital, Shanghai University of Traditional Chinese Medicine, Shanghai, China; ^3^ Department of Emergency, Longhua Hospital, Shanghai University of Traditional Chinese Medicine, Shanghai, China

**Keywords:** hepatocellular carcinoma, *Marsdenia tenacissima* (Roxb.) Moon [Apocynaceae], angiogenesis, vascular endothelial growth factor, patient-derived xenografts

## Abstract

The extract of *Marsdenia* *tenacissima* (Roxb.) Moon [Apocynaceae] (MTE) has shown a significant anti-cancer effect on hepatocellular carcinoma (HCC), but its mechanism remains unclear. In this study, we used transcriptomics methods to investigate the underlying mechanism of MTE against HCC. Both MHCC97H and HepG2 cell lines were treated with MTE. The cell viability and migration were measured using the cell counting kit-8 assay and transwell assay. RNA-sequencing was used to identify differentially expressed genes (DEGs) between HepG2 cells treated with and without MTE. The expression levels of selected DEGs—vascular endothelial growth factor-A (VEGFA), platelet-derived growth factor receptor-β (PDGFRB), and von Willebrand factor (VWF)—were verified by RT-PCR and Western blot. The effect of conditioned medium from HCC cells with MTE treatment (CM-MTE) on blood vessels was observed by tube formation assay of HUVECs and chick chorioallantoic membrane (CAM) assay. A mouse model of HCC patient-derived tumor xenograft (PDX) was established and treated with MTE. The effect of MTE on the growth and angiogenesis of HCC-PDX was analyzed. The results demonstrated that MTE inhibited the viability and migration of HCC cells. RNA-seq showed that MTE treatment downregulated multiple genes associated with metabolism and angiogenesis. The expression levels of VEGFA, VWF, PDGFB, and PDGFRB in HCC cells were significantly suppressed by MTE. Meanwhile, MTE effectively inhibited the tube-forming capability of HUVECs and the angiogenesis of chick CAM. *In vivo* experiments revealed that the extract reduced tumor volume, inhibited the proliferation of HCC cells, and expanded the necrotic area of the tumor. Immunohistochemical results showed that the expression levels of CD31, PDGFB, VEGF, VWF, and PDGFRB in the HCC-PDX tumor tissues were all downregulated by MTE in a dose-dependent manner. Taken together, MTE could inhibit angiogenesis by repressing the expression of VEGF, VWF, PDGF, and PDGFRB in HCC cells, a mechanism that may enable MTE to counter HCC development.

## Introduction

Hepatocellular carcinoma (HCC), a common primary malignant tumor of the liver, causes at least 700,000 deaths annually (McGlynn et al., 2021). HCC usually develops from chronic liver disease, with HBV infection, HCV infection, alcoholism, and non-alcoholic fatty liver diseases as major risk factors (Llovet et al., 2021). Surgical treatments include surgical resection, transarterial chemoembolization (TACE), local radiofrequency ablation (RFA), liver transplantation, and other radical surgeries (Ju and Heimbach, 2020). Non-surgical treatments mainly include chemotherapy, targeted therapy, and immunotherapy (Huang et al., 2020). However, there are still crucial challenges in the treatment of HCC.

In China, traditional Chinese medicine (TCM) has long been used as a supplementary or alternative therapy for HCC (Liu et al., 2020). As an adjuvant therapy, TCM can improve the efficacy of chemotherapy and the quality of life of cancer patients (Guo et al., 2020). Active components in TCM can inhibit the malignant potential of tumor cells (Liu et al., 2020). *Marsdenia tenacissima* (Roxb.) Moon [Apocynaceae] (MT) was incorporated into the Pharmacopoeia of the People’s Republic of China in 2010, known as “Tongguang San” or “Tongguan Teng.” MT, bitter and slightly cold, can be used to deal with pulmonary diseases, cystitis, rheumatism, and anticancer, with few side effects (Chinese Pharmacopoeia Commission, 2015). MT extract (MTE) effectively strengthens the immunity and prolongs the survival of cancer patients (Wang X. et al., 2018). MTE has obvious anti-tumor, hepato-protective, and immunomodulatory effects (Wang P. et al., 2018). It has shown effects on various tumors, including lung cancer (Hu Y. et al., 2020), ovarian cancer (Zheng et al., 2016), and gastric cancer (Li et al., 2020). MTE can induce apoptosis and inhibit autophagy by activating the MEK/ERK pathway in non-small cell lung cancer (Jiao et al., 2018). MTE alleviates mesenteric artery resistance by inhibiting calcium influx and stimulating the phosphorylation of endothelial nitric oxide synthase (eNOS) (Hao et al., 2019). MTE curbs the proliferation and angiogenesis of lymphoma in A20 mice, making it a natural therapeutic drug for lymphoma (Dai et al., 2017). Clinical trials have shown that MTE combined with chemotherapy can enhance the immune response, promote tumor cell apoptosis, and inhibit angiogenesis in locally advanced nasopharyngeal cancer patients (Xu et al., 2020). Binding to S-1, MTE can enhance its inhibitory effect on the proliferation, invasion, and metastasis by reducing the protein levels of VEGFA and MMP-9 expression and affecting the epithelial-to-mesenchymal transition (EMT) pathway (Wen et al., 2021). However, few studies have assessed the efficacy and mechanisms of MTE in treating hepatocellular carcinoma.

In this study, we investigated the biological effect of MTE on hepatoma cell lines and the HCC patient-derived tumor xenograft (PDX) mouse model. We then explored its underlying molecular mechanisms using transcriptomic analysis. Interestingly, we found that MTE may exert its anti-tumor growth effect partially by inhibiting angiogenesis.

## Materials and Methods

### Experimental Drug

MTE (Trade Name: Xiao-Ai-Ping injection) was obtained from Nanjing Sanhome Pharmaceutical Co., Ltd. (Nanjing, China) (Batch No. 202006091M). MTE was prepared as previously reported according to the Drug Standards of the Ministry of Public Health of the Peoples Republic of China (Jiao et al., 2018). Briefly, 1 kg powder of the stem of *Marsdenia tenacissima* (Roxb.) Moon [Apocynaceae] (MT) was extracted with boiling water three times (1.5 h, 1 h, and 0.8 h, respectively). The combined extract was filtered, concentrated, and precipitated with 8 times the volume of 85% ethanol at 4°C for 24 h. The ethanol with extract was recovered thoroughly, and the insoluble precipitate was removed by filtration. Then, the extract was concentrated and added with 0.3% polysorbate 80 and adjusted till pH reached 5.5–6.0 to get 0.2 g/ml MTE solution. The drug was diluted with a culture medium for cell culture or with normal saline for intraperitoneal injection in mice.

### Cell Cultures

Human hepatoma cells (MHCC97H and HepG2) and human umbilical vein endothelial cells (HUVECs) were purchased from the cell bank of the Chinese Academy of Sciences (Shanghai, China). HepG2 and MHCC97H cells were cultured in DMEM with 10% fetal bovine serum (FBS, Lonsera, Grand Island, USA) and HUVECs in endothelial cell medium (ECM) with 5% FBS in an incubator at 37°C and with a humidified atmosphere containing 5% CO_2_. Each experiment was independently performed at least three times.

### CCK-8 Assay

HepG2 and MHCC97H cells were seeded in 96-well plates (3 × 10^3^ cells per well) and incubated at 37°C and 5% CO_2_ for 24 h. The cells were treated with 0, 1, 2, 3, 4, 5, 6, 7, and 8 mg/ml of MTE for 24 h. Then, DMEM containing 10% CCK-8 (Dojindo, Kumamoto, Japan) was added to each well and incubated for 4 h. The absorbance was measured at 450 nm using the microplate reader (BioTek, Winooski, USA).

### Cell Migration Experiment

HepG2 and MHCC97H cells were seeded into the upper chamber of the transwell insert (1 × 10^4^ cells, 200 μL per well) with MTE (0, 1.5, or 3 mg/ml) in DMEM without FBS. DMEM (600 μL per well) containing 10% FBS was added to the lower compartment. After 12 h, the cells were fixed with 4% paraformaldehyde for 30 min. The upper side of the chamber membrane was wiped using a cotton swab. Then, the membrane was stained with 0.1% crystal violet for 20 min and was finally photographed under a 400× microscope. The cells passing through the membrane were counted.

### Transcriptomic Sequencing Analysis

HepG2 cells were treated with 1.5 mg/ml or 3.0 mg/ml MTE for 24 h. The cells were cultured in DMEM as the control group. Total RNA was extracted by TRIzol reagent (Thermo Fisher Scientific, USA), and RNA concentrations were detected by NanoDrop. The RNA sequencing was accomplished by Shanghai Oe Biotech Co., Ltd. (Shanghai, China), mainly including RNA library construction, sequencing data filtering, and comparison. According to the sequencing methods in the literature, genes with a q-value ≤0.05 and fold change ≥2 were defined as differentially expressed genes (DEGs) (Li et al., 2021). Then, the enrichment of the DEGs was analyzed using GO functional enrichment analysis and KEGG pathway analysis.

### RT-PCR

HepG2 cells and MHCC97H cells were seeded in 6-well plates (3 × 10^5^ cells per well) and treated with or without MTE for 24 h. Total RNA was extracted using TRIzol reagent. The extracted total RNA was reverse-transcribed into cDNA using a reverse transcription kit (Applied Biosystems, Carlsbad, USA). The mRNA expression levels of verified genes were detected with an SYBR Green PCR Mix kit (Accurate Biology, Changsha, China) using the StepOnePlus Real-Time PCR System (Applied Biosystems), and β-actin was used as the internal reference for the analysis. The primers were synthesized by ShineGene Biotechnology (Shanghai, China), and the sequences are listed in Table 1.

**TABLE 1:** Sequences of primers used in qPCR.

Gene	Sequence (5′-3′)
h β-actin	Forward: TCA​AGA​AAG​GGT​GTA​ACG​CAA​CTA
	Reverse: CGA​CAG​GAT​GCA​GAA​GGA​GAT
h VWF	Forward: CAA​GAA​ACG​GGT​CAC​CAT​CC
	Reverse: ACC​TCA​AAG​TGA​GTC​TCA​TCC​TTC
h VEGFA	Forward: AAT​CGA​GAC​CCT​GGT​GGA​CA
	Reverse: TGT​TGG​ACT​CCT​CAG​TGG​GC
h PDGFRB	Forward: GAG​TTA​GAA​GAC​TCG​GGG​ACC​TAC
	Reverse: CGA​TGC​AGC​TCA​GCA​AAT​TG
h PCK1	Forward: TGG​AAG​GTT​GAG​TGC​GTC​G
	Reverse: TGT​GTT​CTT​CTG​GAT​GGT​CTT​GA
h FN1	Forward: ACA​GAC​CTA​TCC​AAG​CTC​AAG​TG
	Reverse: AAA​TGT​GAG​ATG​GCT​GTG​GTG
h PDGFB	Forward: CAC​GGT​GAC​GCT​GGA​AGA​C
	Reverse: CGA​GTT​TGG​GGC​GTT​TTG

### Western Blot

HepG2 and MHCC97H cells treated with or without MTE for 24 h were lysed with RIPA buffer (Beyotime Biotechnology), and protein concentrations were quantified by the BCA method. Proteins were separated by SDS-PAGE and blotted onto polyvinylidene fluoride (PVDF) membranes. After blocking, the membranes were incubated with the primary antibodies against VEGFA (A12303, ABclonal, China), PDGFRB (A2180, ABclonal, China), VWF (A13523, ABclonal, China), and the internal reference β-actin (AC026, ABclonal, China) at 4°C overnight. Also, the membrane was then incubated with the secondary antibody for 1 h at room temperature. After being incubated with the ECL-enhanced luminescent substrate, protein expression was detected using a chemiluminescence image analysis system (Tanon-5200, Shanghai, China).

### Tube Formation Assay

The 24-well plate was coated with Matrigel (200 μL per well) and solidified in the incubator at 37°C for 1 h. Then, HUVECs were seeded in the coated 24-well plates (1 × 10^5^ cells per well) and incubated for 4 h. The conditioned medium (CM) from HepG2 or MHCC97H cells without MTE treatment (Control), CM from HepG2 or MHCC97H cells with 1.5 mg/ml MTE treatment (CM-MTE-1.5 mg), CM from HepG2 or MHCC97H cells with 3.0 mg/ml MTE treatment (CM-MTE-3.0 mg), or DMEM (blank) was added to HUVECs and incubated for 24 h. The cells were observed and photographed under an inverted microscope (Olympus-BX53, Japan). ImageJ software was used to calculate the number and length of tube branches.

### Chick Chorioallantoic Membrane Assay

Chicken egg CAM assay has widely been used to study the effects of drugs on angiogenesis *in vivo* by observing the morphological responses of the CAM vasculature. The freshly fertilized chicken eggs were incubated for 8 days, and the eggshells were cracked. DMEM (blank) or control CM, CM-MTE-1.5 mg, and CM-MTE-3.0 mg from HepG2 or MHCC97H cells were added to the allantoic membrane of the chicken embryos, respectively. After 48-h incubation at 37°C, the blood vessels were observed under a microscope, and photographs were taken, according to the processing method of the literature (Kirchner et al., 1996). The efficacy of CM-MTE was evaluated based on the assessment of the vascular morphology and density.

### Establishment of Patient-Derived Xenografts of the HCC Mice Model

Three groups of HCC samples (EHBHKY2014-03-006) were obtained from Shanghai Oriental Hepatobiliary Surgery Hospital. An area (diameter 2–3 cm) without obvious necrosis was cut from the surgical tumor specimen and stored in the pre-cooled RPMI1640 medium containing P/S. Reference was made to establish PDX for liver cancer in the literature (Wu et al., 2020). The tissue block was planted into the groin of BALB/c nude mice (purchased from Shanghai Slack Laboratory Animal Co., Ltd., and bred at 22°C and relative humidity of 50–70%). When the subcutaneous tumor volume reached around 1,000 mm^3^, the tumor tissue was collected and chopped, then re-planted subcutaneously into nude mice.

### *In Vivo* Experiment

The HCC-PDX mice were randomly divided into four groups (*n* = 5), followed by intraperitoneal injection of normal saline (Control) and 5 mg/kg MTE (MTE-L), 10 mg/kg MTE (MTE-M), and 20 mg/kg (MTE-H). Two weeks after treatment, all mice were sacrificed, and the xenografts were collected. Tumor volume was measured by caliper using the formula: volume = length×width^2^×0.5. Then, the tumor tissues were fixed in neutral buffered formalin, embedded in paraffin, and cut into 4-μm sections, followed by H&E staining. All slides were scanned and digitalized, converted to the Hamamatsu’. ndpi file format, and uploaded into HALO software (Version 2.2; Indica Labs, USA) for image analysis.

### Immunohistochemistry

Slices were dewaxed in xylene twice, and then rehydrated in gradient ethanol solution. After antigen recovery using the microwave method in citric acid solution, the sections were blocked in 5% BSA in TBST for 1 h at room temperature, then incubated with the primary antibodies against CD31 (1:250, A19014), VEGFA (1:200), PDGFRB (1:200), VWF (1:200), PDGFB (1:200, A1195), and VEGF (1:200, A16703) purchased from ABclonal (Wuhan, China) and Ki67 (ready-to-use, MAB-0672, Maixinbio, China) at 4°C overnight. The secondary antibody in the streptavidin-biotin kit (#KIT-9720; Maixin-Bio, China) was added to the slides and incubated for 1 h. DAB substrate solution was used to show the positive stain, and the sections were counterstained with hematoxylin. The sections were photographed, and two individuals (G. Y. and Y. C.) independently evaluated all these slides and analyzed the final results using a semiquantitative scoring system as previously described (Gao et al., 2021).

### Statistical Analysis

The data were expressed as mean ± standard deviation (SD) and analyzed using GraphPad Prism 8.0 software. Statistical analysis of the data was performed using the Student’s t-test or analysis of variance (ANOVA) with Tukey’s post-test. *p* < 0.05 was considered to indicate statistically significant differences.

## Results

### MTE Inhibited the Viability and Migration of HCC Cells *In Vitro*

**FIGURE 1:**
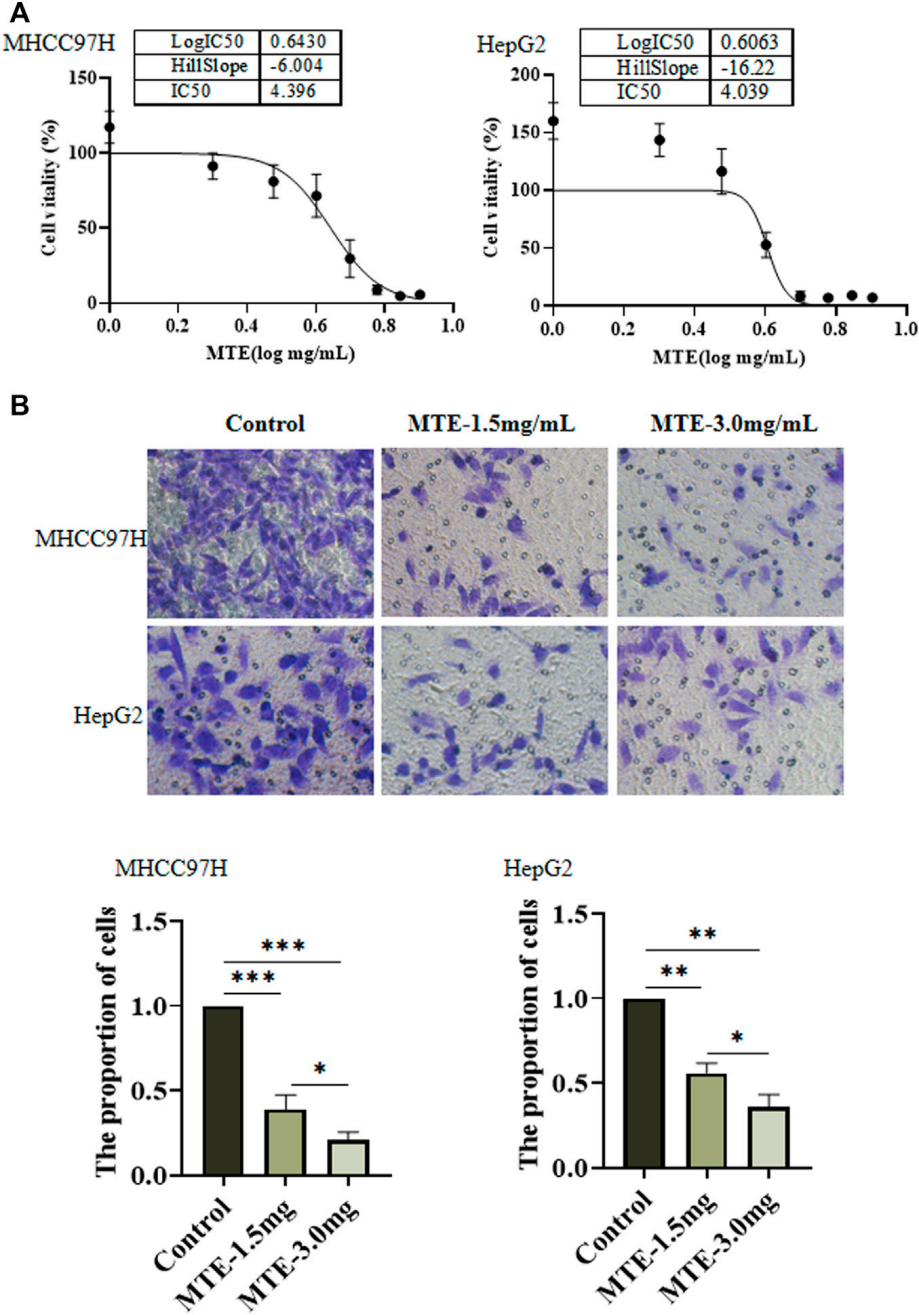
MTE inhibited the viability and migration of HCC cells *in vitro*. **(A)** MHCC97H and HepG2 cells were seeded in 96-well plates and exposed to MTE at different concentrations (0, 1, 2, 3, 4, 5, 6, 7, and 8 mg/ml) for 24 h to assess cell viability by CCK-8 assay. **(B)** Migration potential of MHCC97H and HepG2 cells with or without MTE treatment was measured by transwell assay. **p < 0.01; NS, not significant.

Hepatoma cells were treated with MTE at gradient concentrations from 0 to 8 mg/ml for 24 h and subjected to CCK-8 assay. As shown in Figure 1A, the cell viability was decreased by MTE in a dose-dependent manner. The IC_50_ of MTE was 4.396 and 4.039 mg/ml for MHCC97H and HepG2 cells, respectively (Figure 1A). Also, according to this result, 1.5 mg/ml was set as the low dose and 3.0 mg/mL as the high dose of MTE to treat cells for further experiments.

Figure 1B shows the result of the cell migration experiments. Compared with the control group, the number of cells in the MTE-treated group was significantly reduced (*p* < 0.01), indicating that the ability of cell migration was impaired by MTE.

### Differentially Expressed Genes Associated With Angiogenesis Were Identified

**FIGURE 2:**
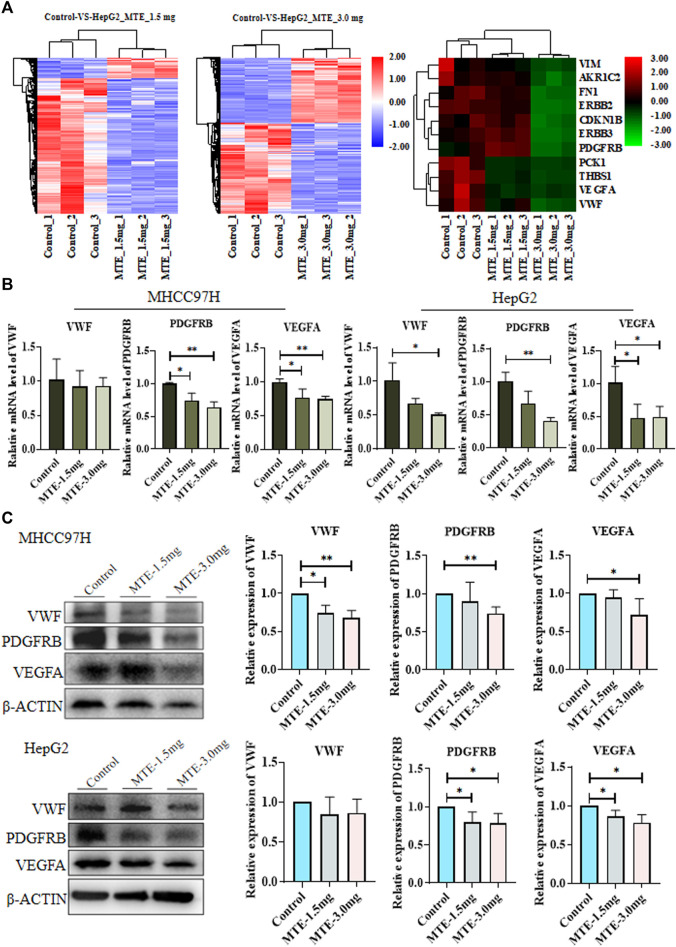
Identification and verification of differentially expressed genes (DEGs) between HCC cells treated with or without MTE. **(A)** HepG2 cells were treated with vehicle, 1.5 mg/ml MTE, or 3.0 mg/ml MTE for 24 h and subjected to RNA-sequencing. The clustering of differentially expressed genes of control vs. MTE-1.5 mg/ml and control vs. MTE-3.0 mg/ml was presented. The DEGs associated with metabolism and angiogenesis were clustered. **(B)** mRNA expression levels of VWF, VEGFR, and PDGFRB in MHCC97H and HepG2 cells treated with or without MTE. **(C)** Protein levels of VWF, VEGFR, and PDGFRB in HCC cells treated with or without MTE. *p < 0.05; **p < 0.01.

RNA-seq screened 3,664 DEGs between cells treated with or without MTE, including 87 upregulated and 590 downregulated in the MTE low-dose group, and 1,422 up-regulated and 1,565 down-regulated in the MTE high-dose group. These DEGs were enriched in tumor-related signaling pathways, such as cell cycle, apoptosis, angiogenesis, and metabolism. We noticed that many genes were associated with angiogenesis and metabolism signaling, including VWF, VEGFA, PDGFRB, PCK1, fibronectin (FN), vimentin (VIM), AKR1C2, ERBB2, ERBB3, THBS1, and CDKN1B (Figure 2A).

The expression of part of these genes was further verified by RT-PCR analysis. The results showed that the mRNA expression levels of VWF, PDGFRB, and VEGFA were significantly reduced by MTE in both HCC cells dose dependently, except that the mRNA expression of VWF had a decreasing trend in MHCC97H cells. The expression of PDGFB, an important angiogenic cytokine, was also detected and showed down-regulated by MTE dose-dependently in both HCC cells. The expression of other selected genes showed no obvious change between the MTE group and the control (Figure 2B and Supplementary Figure S1).

Then, the protein expression levels of VWF, PDGFRB, and VEGFA in HCC cells were further measured by Western blot. Compared with those in the control group, the protein levels of the three genes in the MTE-3.0 mg/ml group all decreased remarkably in MHCC97H cells. In addition, the expression levels of PDGFRB and VEGFA were downregulated significantly in HepG2 cells. There was a decreasing trend in VWF when treated with MTE (Figure 2C). Therefore, these data suggested that MTE could regulate the expression of angiogenesis-related molecules in HCC cells.

### MTE Inhibited Tumor-Related Angiogenesis

Tumor angiogenesis is necessary for tumor growth, invasion, and metastasis (Jiang et al., 2020). Considering the essential role of VEGFA, PDGFRB, and VWF in angiogenesis, we conducted the tube formation assay and chick CAM experiments to study the potential effect of MTE on angiogenesis.

**FIGURE 3:**
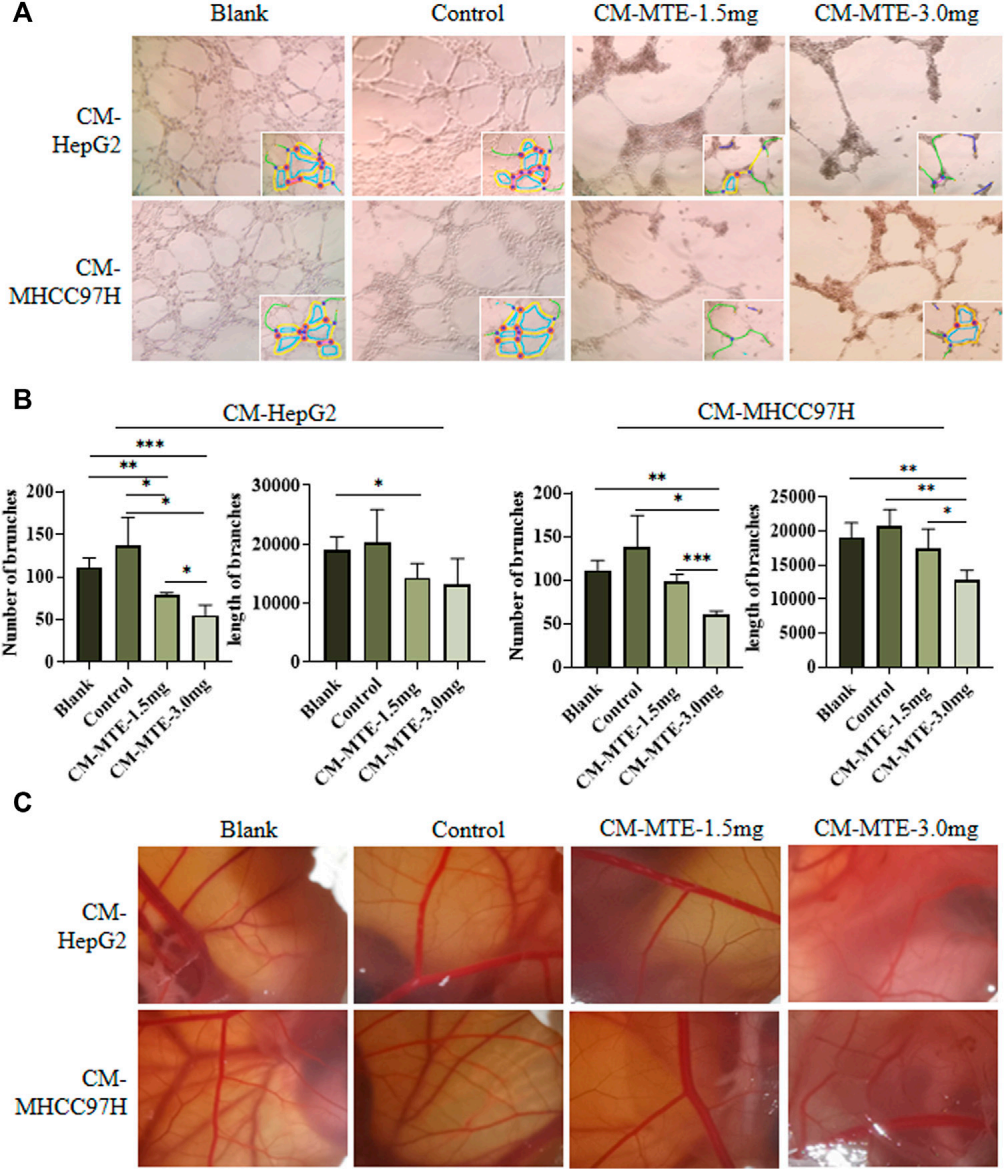
MTE inhibited tumor angiogenesis by regulating the expression of pro-angiogenesis molecules in HCC cells. **(A)** Effect of conditioned medium (CM) from HCC cells treated with or without MTE on endothelium tube formation was detected by tube formation assay of HUVECs. **(B)** Statistical analysis of the number and length of endothelium tube branches in different groups. **(C)** Effect of CM from HCC cells treated with or without MTE on angiogenesis was detected by chick CAM assay. Blank: DMEM; control: CM from HepG2 or MHCC97H cells without MTE treatment; CM-MTE-1.5 mg: CM from HepG2 or MHCC97H cells treated with 1.5 mg/ml MTE; CM-MTE-3.0 mg: CM from HepG2 or MHCC97H cells treated with 3.0 mg/ml MTE. *p < 0.05; **p < 0.01; ***p < 0.001.

In the tube formation assay of HUVECs, statistical analysis showed a significant increase in the number and length of branches in the CM-control group of both HepG2 and MHCC97H cells, compared to those in the blank group. CM-MTE from HepG2 and MHCC97H attenuated the number of branches of HUVECs in a dose-dependent manner. Meanwhile, the CM-MTE from MHCC97H cells significantly reduced the length of branches dose dependently (Figures 3A,B). Thus, MTE could inhibit the formation of blood vessels by reducing pro-angiogenesis molecules in the culture medium of HCC cells.

The results of the chick CAM assay to observe neovascularization are presented in Figure 3C. The new blood vessels of CAM in the blank group (only DMEM) grew well and branched moderately. Compared with the blank group, vascular growth was vigorous with more branches in the CAM incubated with the control CM from HepG2 and MHCC97H cells. After MTE treatment, the number and branches of blood vessels decreased.

### MTE Increased the Tumor Necrotic Area and Inhibited Tumor Growth in HCC-PDX Mice

**FIGURE 4:**
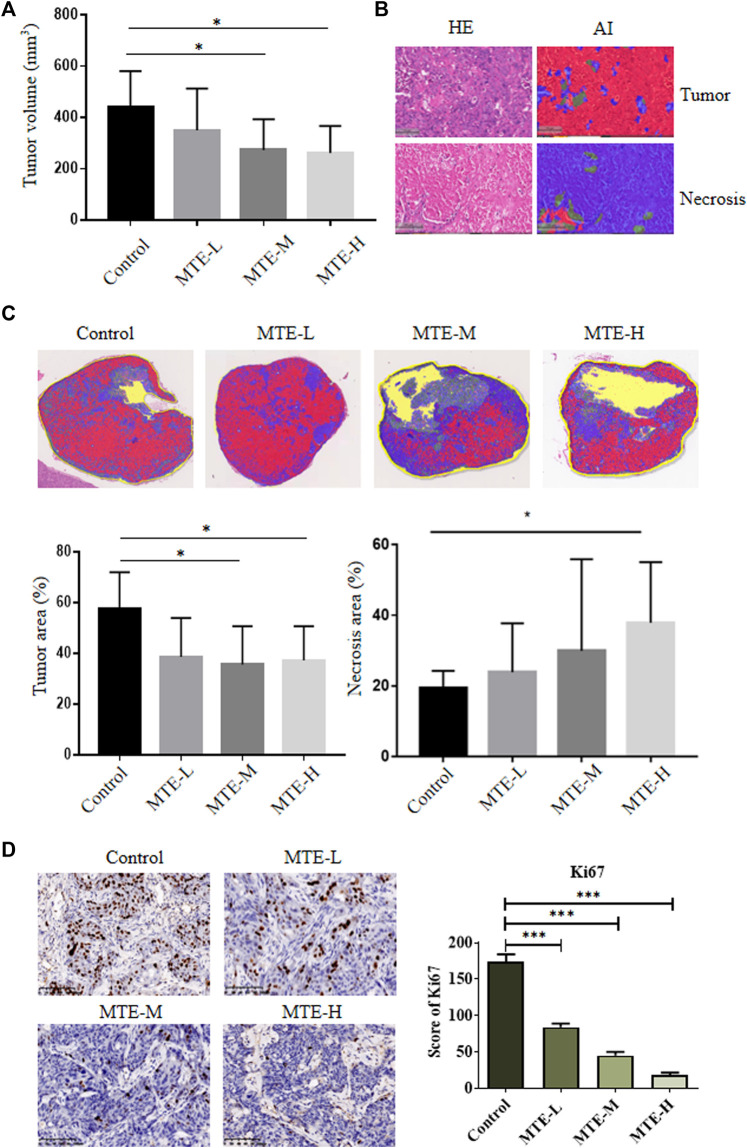
MTE increased the tumor necrotic area and inhibited tumor growth of HCC-PDXs. **(A)** HCC-PDXs were treated with different doses of MTE (L: 5 mg/kg; M: 10 mg/kg; H: 20 mg/kg) for 2 weeks, and tumor volumes were measured. **(B)** AI techniques were used to display the tumor area (red) and necrotic area (blue). Magnification of HE: ×100. **(C)** Representative whole-slide images of the tumor labeled by AI algorithm and the percentage of tumor cells and necrotic area are plotted. **(D)** Representative images of Ki67 staining on slides of HCC-PDXs. Magnification of IHC: ×200. HE, hematoxylin and eosin; AI, artificial intelligence. *p < 0.05. ***p < 0.001.

Compared with the control group, the volume of tumors in the MTE-treated groups was significantly decreased (Figure 4A).

Given the extensive necrosis in the HE-stained whole-slide images (WSIs), artificial intelligence (AI) technology was used to display viable (red) or necrotic (blue) tumor cells (Figure 4B). The tumor area accounted for about 60% of tissues in the control group and about 40% in the treatment group (Figure 4C). The necrotic area accounted for about 20% of tissues in the control group and about 40% in the MTE-H group (Figure 4C).

We also detected the expression of Ki67 in these PDX specimens and found that the expression level of Ki67 was significantly downregulated in a dose-dependent manner (Figure 4D).

### MTE Inhibited Tumor Angiogenesis in HCC-PDX Mice

**FIGURE 5:**
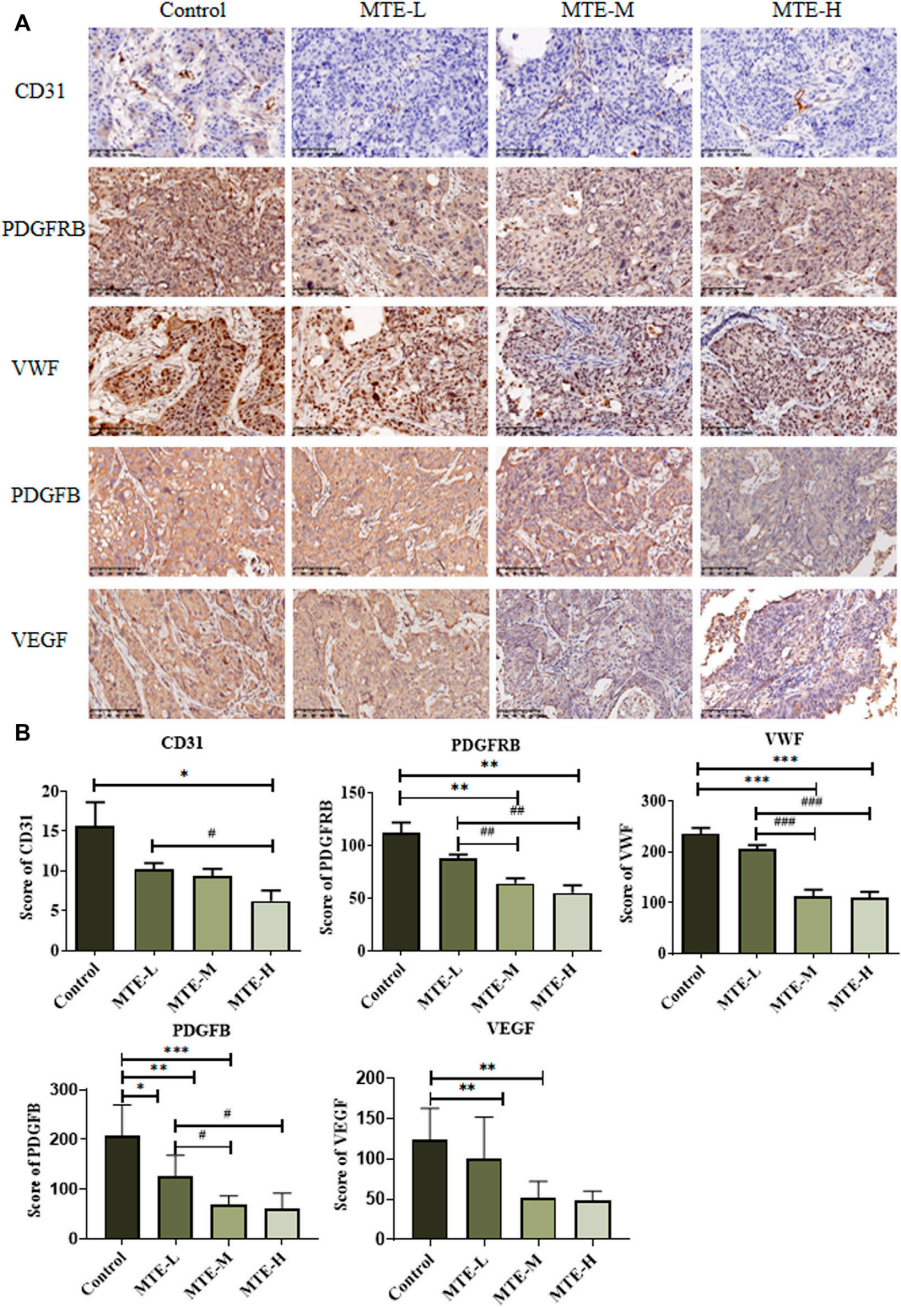
MTE inhibited tumor angiogenesis in the HCC-PDX mouse model. **(A)** Representative images of IHC of angiogenesis-associated proteins (CD31, PDGFRB, VWF, PDGFB, and VEGF) on pathological slides of HCC-PDX tissues from control, MTE-L, MTE-M, and MTE-H groups. **(B)** Score of CD31, PDGFB, VEGF, PDGFRB, and VWF positive staining in these slides. Magnification: ×200. Compared with control, *p < 0.05; **p < 0.01; ***p < 0.001. Compared with MTE-L, ^#^ *p* < 0.05; ^##^ *p* < 0.01; ^###^ *p* < 0.001.

The aforementioned *in vitro* results proved that MTE could inhibit angiogenesis by targeting some angiogenesis-related biomarkers. We then detected the protein expression of PDGFRB, VWF, PDGFB, VEGF, and CD31 in PDX specimens. The expression levels of PDGFRB and VWF were significantly reduced in the MTE-treated group dose dependently, which was consistent with the *in vitro* results. We also found that PDGFB, VEGF, and CD31 expression levels were also significantly downregulated by MTE intervention (Figures 5A,B).

## Discussion

Previous studies have shown the effects of MTE on some tumors. However, most studies focused on the cytotoxic effect of MTE on tumor cells, especially their apoptosis and cell cycle (Zheng et al., 2016), but rarely on angiogenesis in HCC. Tumor angiogenesis, crucial for tumor growth and distant metastasis, is controlled by pro-angiogenic and anti-angiogenic cytokines, contributing to tumor angiogenesis. Pro-angiogenic factors (such as VEGF and PDGF) secreted by cancer cells can stimulate angiogenesis of endothelial cells by the paracrine mechanism, thus contributing to tumor neovascularization (Lázár-Molnár et al., 2000). The formation of a vascular network within the tumor is necessary to provide nutrition for tumor growth and metastasis. In the present study, we found that MTE downregulated the expression of some angiogenic factors (PDGFB, VEGFA, and VWF). The abnormal secretion of pro-angiogenic factors leads to the formation of an abnormal vascular network, which enhances the aggressiveness of the tumor and makes it more likely to escape from internal immunity and external treatment (Viallard and Larrivée, 2017). These growth factors mainly include VEGF, PDGF, and FGF2 (Wei et al., 2020). It is noteworthy that VEGFA plays a dual role in angiogenesis, either promotive or inhibitive (Zhao et al., 2019). Prospective multicenter studies have found that serum VWF levels can influence HCC recurrence, and higher VWF is associated with early recurrence of hepatocellular carcinoma (Aryal et al., 2019). Interestingly, there are few clues that MTE inhibits angiogenesis by downregulation of VEGF signaling previously (Wang et al., 2016; Dai et al., 2017). The molecular network profiling and multi-omic joint analyses using an H22 mouse model of HCC have suggested that MTE might inhibit angiogenesis by targeting HIF1α (Li et al., 2022).

To study the effect of MTE on tumor angiogenesis, tube formation assay of HUVECs and CAM experiments were performed. We found that the formation of blood vessels was inhibited by the conditioned culture medium of HCC cells treated with MTE. These findings suggest that MTE can affect tumor angiogenesis by repressing the expression of angiogenic molecules such as VEGF, VWF, and PDGFRB, a mechanism that counters the HCC development.

Based on the anti-tumor potential of MTE *in vitro*, we established a PDX model to further confirm its anti-tumor efficacy *in vivo*. Compared with the traditional cell-derived xenograft (CDX) model previously used to evaluate the effect of MTE *in vivo*, the PDX model is much valuable because it maintains the genetic characteristics and heterogeneity of the primary tumor (Hu B. et al., 2020). In the present study, the tumor volume was found inhibited by MTE. We then invoked the AI technique to display the status inside the tumor tissues and found that after MTE administration, the area of tumor necrosis increased in a dose-dependent manner. This characteristic has been observed in the treatment with targeted therapy—the tumor size does not change much, but necrosis or liquefaction exists in the interior (Xu, 2010). In this study, IHC staining of CD31 showed that blood vessels were reduced by MTE. Also, the angiogenesis-related molecules including PDGFB, VEGF, VEGFA, and PDGFRB were significantly downregulated by MTE, especially by high-dose MTE in the HCC-PDX tumor. These results further support the effect of MTE on angiogenesis and the related factors. Inhibition of angiogenesis blocks blood supply, which may eventually lead to necrosis of tumor tissues. Therefore, MTE retards tumor growth partially by targeting tumor angiogenesis.

In summary, the valuable finding of this study is that MTE, an extract of a traditional Chinese medicinal herb, could retard the growth of HCC by targeting angiogenesis. In detail, MTE exerted its anticancer potential via downregulating angiogenesis-related biomarkers, including VEGFA, PDGFRB, and VWF. Moreover, by blocking the secreted angiogenesis molecules, MTE indirectly impeded vascular network generation in the tumors.

These novel findings demonstrate a unique property of MTE as an herb agent for cancer treatment. Given the association between angiogenesis and bloody ascites, MTE is supposed to inhibit the formation of ascites, which has been proved by our ongoing studies and previous report (Shi et al., 2011). However, one should be cautious in prescribing MTE to treat pediatric or pregnant patients. Furthermore, studies are needed to verify the mechanism of MTE in regulating the expression of VEGF, PDGF, PDGFRB, and VWF, which will promote MTE to be used as a useful alternative clinical treatment for HCC.

## Data Availability

The datasets presented in this study can be found in online repositories. The names of the repository/repositories and accession number(s) can be found at: https://www.ncbi.nlm.nih.gov/bioproject/, PRJNA795717.

## Supplementary Material

Supplementary Figure S1 mRNA expression levels of some selected DEGs in HCC cells treated with or without MTE. The mRNA expression levels of PDGFB, PCK1 and FN1 in MHCC97H and HepG2 cells were treated with or without different doses of MTE. **P* < 0.05.

## Abbreviations

AI, artificial intelligence; CM, conditioned medium; CAM, chorioallantoic membrane; DEGs, differentially expressed genes; HCC, hepatocellular carcinoma; HUVECs, human umbilical vein endothelial cells; IHC, immunohistochemistry; MTE, extract of *Marsdenia tenacissima* (Roxb.) Moon [Apocynaceae]; PDX, patient-derived tumor xenograft; PDGFB, platelet-derived growth factor B; PDGFRB, platelet-derived growth factor receptor-β; VEGFA, vascular endothelial growth factor-A; VWF, von Willebrand factor.
