# Supplementary figures and images for: Extract of Marsdenia tenacissima (Roxb.) Moon [Apocynaceae] Suppresses Hepatocellular Carcinoma by Inhibiting Angiogenesis
Source: Front Pharmacol. 2022 Jun 30;13:900128. doi: 10.3389/fphar.2022.900128 (PMC9279733; doi:10.3389/fphar.2022.900128)

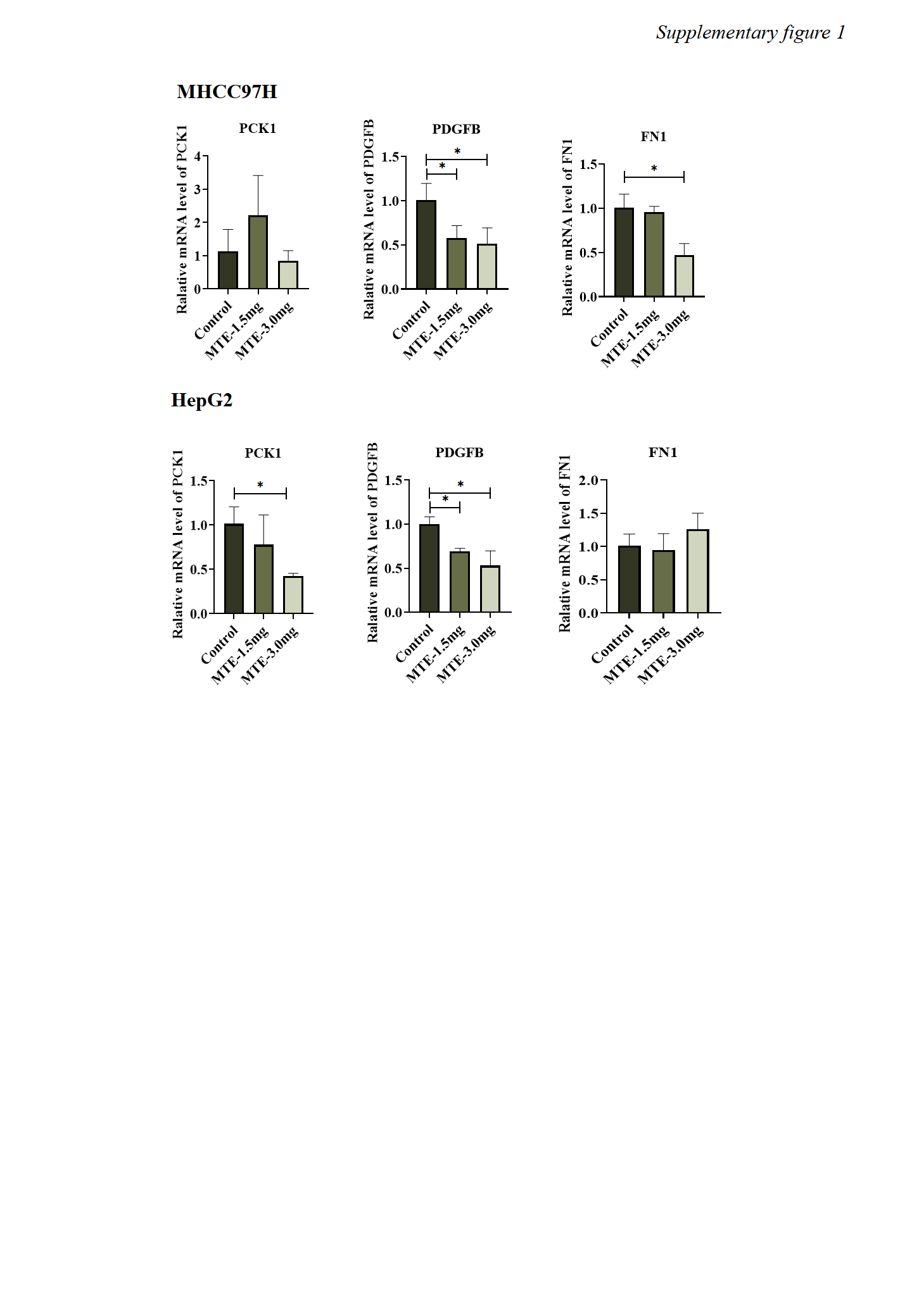

Supplement: Supplementary file 1 [file Image1.tif]
